# Supplementary material for: Mass Spectrometry-Based Proteomics for Discovering Salivary Biomarkers in Periodontitis: A Systematic Review
Source: Int J Mol Sci. 2023 Sep 27;24(19):14599. doi: 10.3390/ijms241914599 (PMC10572407; doi:10.3390/ijms241914599)
Supplement: Supplementary file 1 [file ijms-24-14599-s001.zip › ijms-2560461-supplementary.pdf]

## 2. Materials and Methods

### *2.3. Literature search*

**Table S1.** Literature search strategies.

| **Databases (Start date)** | **Search strategies (Search end date: April 31, 2023)** |
|---|---|
| PubMed (1946) | (((("saliva"[MeSH Terms]) OR ("saliva"[All Fields])) OR ("salivary"[All Fields])) **AND** ((((((("proteomics"[MeSH Terms]) OR ("proteome"[MeSH Terms])) OR ("mass spectrometry"[MeSH Terms])) OR ("proteomics"[All Fields])) OR ("proteomic"[All Fields])) OR ("proteome"[All Fields])) OR ("mass spectrometry"[All Fields]))) **AND** (("periodontitis"[MeSH Terms]) OR ("periodontitis"[All Fields])) |
| Web of Science (1956) | (((TS=(proteomics)) OR TS=(proteome)) OR TS=(mass spectrometry)) **AND** TS=(periodontitis) **AND** ((TS=(saliva)) OR TS=(salivary)) |

**Table S2.** Case and control definition for all shortlisted case-control studies (n = 13).

| Study | Case | Control |
|---|---|---|
| Wu et al. 2009 [32] | **AP:** Age < 30 years; ≥ 3 teeth affected in addition to first molar and incisor; generalized interproximal attachment loss on affected teeth. | < 10% BOP; PPD < 3 mm; < 1% of sites with CAL > 2mm; no radiographic bone loss. |
| Gonçalves et al. 2010 [39] | **CP:** > 35 years; ≥ six sites on different teeth with BOP, PPD > 6 mm, and CAL > 5 mm. | < 10% BOP; PPD < 3 mm. |
| Kim et al. 2010 [40] | **AP:** CAL > 5 mm in ≥ 8 teeth; ≥ 3 teeth affected in addition to first molar and incisor.<br>**CP:** CAL > 5 mm with BOP in all four quadrants; no evidence of rapid progression. | No evidence of CAL, BOP, or radiographic bone loss. |
| Salazar et al. 2013 [33] | **CP:** BOP > 10%; PPD ≥ 5 mm at ≥ 2 sites; PPD ≥ 4 mm at ≥ 40% teeth. | BOP < 30%; maximum PPD ≤ 3 mm. |
| Mertens et al. 2018 [41] | American Academy of Periodontology classification criteria, no details reported. | |
| Bostanci et al. 2018 [42] | **AP:** CAL > 5 mm and PPD > 6 mm in ≥ 8 teeth; ≥ 3 teeth affected in addition to first molar and incisor.<br>**CP:** CAL > 5 mm and PPD > 6 mm in ≥ 4 non-adjacent teeth; > 50% radiographic bone loss in ≥ two quadrants. | No sites with PPD > 3 mm and CAL > 2 mm; no evidence of radiographic bone loss. |
| Grant et al. 2019 [43] | **Periodontal disease:** Radiographic bone loss ≥ 1/3 of the root length and/or PPD > 5 mm, and BOP > 20%. | No tooth with PPD > 5 mm or Radiographic bone loss ≥ 1/3 of the root length. |
| Tang et al. 2019 [44] | **CP:** CAL ≥ 1 mm; PPD ≥ 4 mm; radiographic bone lone; > 30% of teeth involved. | No sites with CAL or PPD > 3 mm; BOP ≤ 20%; no radiographic bone loss. |
| Shin et al. 2019 [45] | **Periodontitis:** Presence of proximal bone of ≥ 3 mm in ≥ 2 non-adjacent teeth. | No proximal bone of ≥ 3 mm in ≥ 2 non-adjacent teeth. |
| Hartenbach et al. 2020 [46] | **CP:** > 10% of teeth with PPD and/or CAL ≥ 5 mm and BOP. | < 10% sites with BOP, no PPD or CAL > 3 mm; < 5% sites of PPD or CAL = 4 mm without BOP was allowed. |
| Antezack et al. 2020 [47] | **Periodontitis:** Inter-dental CAL detectable at ≥ 2 non-adjacent teeth or buccal or oral CAL ≥ 3 m with PPD ≥ 3 mm at ≥ 2 teeth. | BOP < 10%; PPD < 3 mm; no CAL. |
| Grant et al. 2022 [27] | **Birmingham cohort:**<br>Stage I/II periodontitis: interproximal CAL of 2-4 mm at > 8 teeth with PPD of 5-7 mm; Stage III/IV: interproximal CAL > 5 mm at 12 teeth and PPD > 7 mm.<br>**Newcastle cohort:** | **Birmingham cohort:**<br>No sites of interproximal CAL; no sites with PPD > 3 mm; < 10% sites with GI of 1 and no sites with GI of 2 or 3; < 10% sites with BOP. |

| | | |
|---|---|---|
| | Stage I/II periodontitis: interproximal PPD of 5-7 mm (equating to approximately 2-4 mm CAL) at ≥ 8 teeth; Stage III/IV: interproximal PPD > 7 mm (equating to approximately ≥ 5 mm CAL) at ≥ 12 teeth, BOP scores > 30%. | **Newcastle cohort:** No sites with interproximal CAL; PPD ≤ 3 mm in all sites (but would allow up to four 4 mm pockets at distal of last standing molars); ≤ 10% sites with mGI of ≥ 2.0; < 10% sites with BOP. |
| Casarin et al. 2023 [34] | < 35 years; PPD ≥ 5 mm at ≥ 8 teeth and PPD ≥ 7 mm at ≥ 2 teeth; CAL > 5 mm in three teeth other than the first molars and incisors. | No history of CAL, PPD < 4 mm, no radiographic bone loss, ≥ 20 teeth present. |

AP: aggressive periodontitis; BOP: bleeding on probing; CAL: clinical attachment loss; CP: chronic periodontitis; GI: gingival index; PPD: probing pocket depth.

International Journal of *Molecular Sciences*

MDPI

*2.5. Risk of bias assessment*

**Table S3.** Quality assessment by the Newcastle Ottawa Scale for all shortlisted case-control studies (n = 13).

| Study | Selection | | | | Comparability | Exposure | | Total score |
|---|---|---|---|---|---|---|---|---|
| | Case definition | Representativeness of the cases | Selection of controls | Definition of controls | | Ascertainment of exposure | Non-response rate | |
| Wu et al. 2009 [32] | * | * | * | * | * | | * | 6 |
| Gonçalves et al. 2010 [39] | * | | * | * | * | | * | 6 |
| Kim et al. 2010 [40] | * | | * | * | ** | | * | 6 |
| Salazar et al. 2013 [33] | * | * | * | * | ** | | * | 7 |
| Mertens et al. 2018 [41] | * | * | * | * | * | | * | 6 |
| Bostanci et al. 2018 [42] | * | | * | * | * | | * | 5 |
| Grant et al. 2019 [43] | * | | * | * | * | | * | 5 |
| Tang et al. 2019 [44] | * | * | * | * | * | | * | 6 |
| Shin et al. 2019 [45] | * | * | * | * | ** | | * | 7 |
| Hartenbach et al. 2020 [46] | * | | * | * | * | | * | 5 |
| Antezack et al. 2020 [47] | * | * | * | * | * | | * | 6 |
| Grant et al. 2022 [27] | * | | * | * | ** | | * | 6 |
| Casarin et al. 2023 [34] | * | | * | * | ** | | * | 6 |

**Table S4.** Risk of bias assessment by QUADAS-2 for all shortlisted diagnostic studies (n = 13).

| Study | Risk of bias | | | |
|---|---|---|---|---|
| | Patient selection | Index test | Reference standard | Flow and timing |
| Wu et al. 2009 [32] | High | High | Low | Low |
| Gonçalves et al. 2010 [39] | High | High | Low | Low |
| Kim et al. 2010 [40] | High | High | Low | Low |
| Salazar et al. 2013 [33] | High | High | Low | Low |
| Mertens et al. 2018 [41] | High | High | Low | Low |
| Bostanci et al. 2018 [42] | High | Low | Low | Low |
| Grant et al. 2019 [43] | High | High | Low | Low |
| Tang et al. 2019 [44] | High | High | Low | Low |
| Shin et al. 2019 [45] | High | High | Low | Low |
| Hartenbach et al. 2020 [46] | High | High | Low | Low |
| Antezack et al. 2020 [47] | High | Low | Low | Low |
| Grant et al. 2022 [27] | High | High | Low | Low |
| Casarin et al. 2023 [34] | High | High | Low | Low |

**Table S5.** Quality assessment by NIH quality assessment tool for before-after (pre-post) studies with no control group (n = 2).

| Study | Q1 | Q2 | Q3 | Q4 | Q5 | Q6 | Q7 | Q8 | Q9 | Q10 | Q11 | Q12 | Total score |
|---|---|---|---|---|---|---|---|---|---|---|---|---|---|
| Haigh et al. 2010 [48] | Y | Y | N | Y | N | Y | Y | N | Y | Y | N | NA | 7 |
| Yuan et al. 2022 [49] | Y | Y | N | Y | N | Y | Y | N | Y | Y | N | NA | 7 |

N: no; NA; not applicable; NIH, National Institutes of Health; Y: yes.
**Q1**. Was the study question or objective clearly stated?
**Q2.** Were eligibility/selection criteria for the study population prespecified and clearly described?
**Q3.** Were the participants in the study representative of those who would be eligible for the test/service/intervention in the general or clinical population of interest?
**Q4.** Were all eligible participants that met the prespecified entry criteria enrolled?
**Q5.** Was the sample size sufficiently large to provide confidence in the findings?
**Q6.** Was the test/service/intervention clearly described and delivered consistently across the study population?
**Q7.** Were the outcome measures prespecified, clearly defined, valid, reliable, and assessed consistently across all study participants?
**Q8.** Were the people assessing the outcomes blinded to the participants' exposures/interventions?
**Q9.** Was the loss to follow-up after baseline 20% or less? Were those lost to follow-up accounted for in the analysis?
**Q10.** Did the statistical methods examine changes in outcome measures from before to after the intervention? Were statistical tests done that provided p values for the pre-to-post changes?
**Q11.** Were outcome measures of interest taken multiple times before the intervention and multiple times after the intervention (i.e., did they use an interrupted time-series design)?
**Q12.** If the intervention was conducted at a group level (e.g., a whole hospital, a community, etc.) did the statistical analysis take into account the use of individual-level data to determine effects at the group level?

**Table S6.**. Differentially expressed (descending order of absolute fold change) unstimulated or stimulated whole salivary proteins detectable by mass spectrometry-based proteomics.
